# A Synthesizing Semantic Characteristics Lung Nodules Classification Method Based on 3D Convolutional Neural Network

**DOI:** 10.3390/bioengineering10111245

**Published:** 2023-10-25

**Authors:** Yanan Dong, Xiaoqin Li, Yang Yang, Meng Wang, Bin Gao

**Affiliations:** Faculty of Environment and Life, Beijing University of Technology, Beijing 100124, China; dongyanan@emails.bjut.edu.cn (Y.D.); 13011013839@163.com (Y.Y.); mikew@emails.bjut.edu.cn (M.W.); gaobin@bjut.edu.cn (B.G.)

**Keywords:** lung nodule classification, convolutional neural network, multi-view, interpretability, attention mechanism

## Abstract

Early detection is crucial for the survival and recovery of lung cancer patients. Computer-aided diagnosis system can assist in the early diagnosis of lung cancer by providing decision support. While deep learning methods are increasingly being applied to tasks such as CAD (Computer-aided diagnosis system), these models lack interpretability. In this paper, we propose a convolutional neural network model that combines semantic characteristics (SCCNN) to predict whether a given pulmonary nodule is malignant. The model synthesizes the advantages of multi-view, multi-task and attention modules in order to fully simulate the actual diagnostic process of radiologists. The 3D (three dimensional) multi-view samples of lung nodules are extracted by spatial sampling method. Meanwhile, semantic characteristics commonly used in radiology reports are used as an auxiliary task and serve to explain how the model interprets. The introduction of the attention module in the feature fusion stage improves the classification of lung nodules as benign or malignant. Our experimental results using the LIDC-IDRI (Lung Image Database Consortium and Image Database Resource Initiative) show that this study achieves 95.45% accuracy and 97.26% ROC (Receiver Operating Characteristic) curve area. The results show that the method we proposed not only realize the classification of benign and malignant compared to standard 3D CNN approaches but can also be used to intuitively explain how the model makes predictions, which can assist clinical diagnosis.

## 1. Introduction

Lung cancer is the malignant tumor with the highest incidence and mortality rate, and studies have shown that the number of people dying from lung cancer in 2030 will continue to increase, China could reach 42.7% [1,2]. Lung cancer is often overlooked in the early stage due to the lack of obvious symptoms [3], and when detected, it is usually in the advanced stage, often accompanied by multiple organ or lymph node metastasis, which makes the treatment difficult and ineffective. Early lung cancer is mostly manifested as lung nodules, which can be treated with surgery and radiotherapy, so early screening is of great significance to the prevention and treatment of lung cancer [4]. Studies have shown that low-dose spiral CT (Low-dose Computed Tomography (LDCT)) screening methods can significantly improve lung cancer detection rates and reduce the morbidity and mortality of people at a high risk of lung cancer by 20% compared to chest radiography screening methods [5].

The popularization of LDCT technology has achieved a high yield of CT images. Consequently, the workload of manual reading has increased dramatically [6], which not only burdens doctors with time-consuming and cumbersome work but also may cause fatigue misdiagnosis and omission of diagnosis [7]. For this reason, researchers have proposed a computer-aided diagnosis (CAD) system for lung nodule classification to improve the effectiveness of diagnosis [8]. In recent years, CAD systems have been widely used to assist in the treatment of different diseases due to their efficiency and reliability in clinical diagnosis [9,10]. Benign and malignant identification is the top priority in the auxiliary diagnosis of lung nodules. Generally speaking, the traditional computer-aided diagnosis system extracts the underlying features of the image from the candidate nodules after lung parenchyma segmentation and adopts traditional classifiers for learning. In contrast, with the rise of deep learning technology, its application in medical images has gradually become a mainstream trend [11].

Deep learning techniques create a form of end-to-end automated processing that integrates feature selection and extraction in a single architecture, significantly improving efficiency and accuracy [12]. Shen et al. [13] proposed MC-CNN (Multi-crop convolutional neural networks) to simplify the traditional way of classifying the malignancy of lung nodules by using convolutional neural networks to learn the features generated at multiple scales, effectively reducing computational complexity. Liu et al. [14] designed multiple independent neural networks to simulate different expert behaviors and fused the results with integrated learning, consisting of three different types of architectures to form a multi-model 3D CNN. Ciompi et al. [15] extracted multi-view features of lung nodules by analyzing three views, axial, coronal and sagittal, and constructed a multi-scale representation using three scales, which is more conducive to the classification of lung nodules. Zheng et al. [16] proposed a deep convolutional neural network, STM-Net (Scale-Transfer Module Net), which contains a scale-shifting module and multi-feature fusion operation, and the model adapts to the size of the target by scaling the images with different resolutions.

Although convolutional neural networks can simplify complex processing steps, some problems inevitably arise. Networks are often characterized by complex parameters, layers and high dimensionality of data, making it difficult to intuitively understand how they make decisions and unable to explain the logic behind their predictions. They are often considered as a “black box” [17]. Especially in the medical field [18], it is necessary to have a clear explanation and basis for the model’s judgment in order to help radiologists make more profound diagnoses.

When doctors assess lung nodules with CT images, they use characteristics such as lobulation, texture, diameter, subtlety and degree of calcification to describe and analyse their relevant manifestations [19]. These semantic characteristics are also often found in radiology reports. Clinically speaking, these semantic characteristics are important reference factors for determining the benignity and malignancy of pulmonary nodules and correlate with each other [20]. Utilizing shared features among multiple semantic characteristics can achieve mutual enhancement between features. Studies have shown that multi-task learning can improve performance when similar domain background tasks are involved [21]. Wu et al. [22] designed PN-SAMP (Pulmonary Nodule Segmentation Attributes and Malignancy Prediction) to combine lung nodule segmentation, semantic characteristics and benign and malignant prediction, which helps improve a single task’s performance. Zhao et al. [23] proposed a multi-scale multi-task combined 3D CNN that can detect benign and malignant lung nodules from CT scan Classification. This CNN combined two image features of different volume scales, followed by multi-task learning to achieve benign, malignant and semantic feature classifications of lung nodules. Li et al. [24] incorporated domain knowledge into the CNN and achieved the classification of nine semantic characteristics of lung nodules by multi-task learning with improved overall performance of the model.

**Figure 1:**
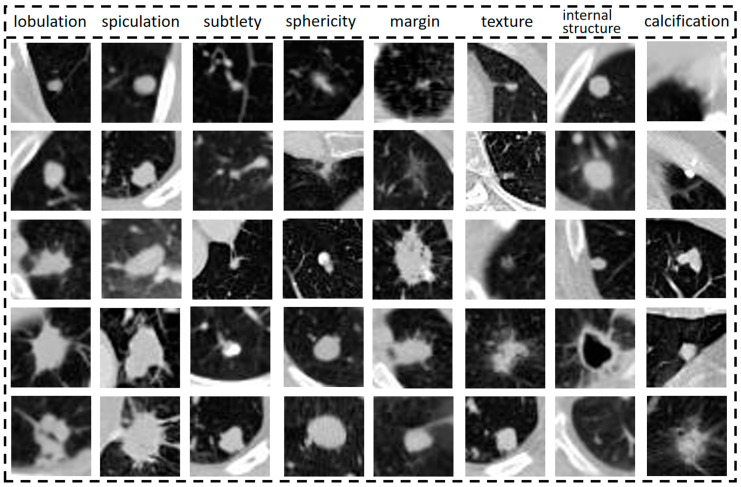
Semantic characteristic performance of lung nodules.

The performance of each semantic characteristic common to lung nodules is shown in Figure 1, from top to bottom, indicating the elevated level of semantic characteristics represented by the column. Semantic characteristics are more intuitive and informative for clinicians [25], so this study synthesizes them to assist in benign and malignant diagnosis and analysis. Semantic characteristics are intuitive to radiologists and provide objective methods to capture image information. There is an opportunity to incorporate these semantic characteristics into the design of deep learning models, combining the best of both worlds.

Similarly, the diagnostic process focuses on different semantic characteristics by repeatedly observing them. Attention mechanisms proposed in recent years also draw on the humans ability to focus attention when processing information [26]. In computer vision tasks, by assigning different weights to the input image, the model is allowed to selectively focus on a specific portion of the input to understand the data better. Zhang et al. [27] designed the LungSeek model that combines the SK-Net with a residual Network to simultaneously extract lung nodule features from both spatial and channel dimensions, improving lung nodules detection effectiveness. Fu et al. [28] added an attention module to the model, which can compute the importance of each slice to filter out irrelevant slices. AI-Shabi et al. [29] proposed a 3D axial attention, applying the attention to each axis individually, thus providing complete 3D attention to focus on the nodes efficiently. There are not many studies that take semantic characteristics and attention mechanisms into account, but simply classify malignant labels. We try to incorporate this domain knowledge into a deep learning framework, and the mentioned studies are summarized in Table 1.

In this paper, we design a convolutional neural network (SCCNN) with semantic characteristics and integrate the multi-view, multi-tasking and attention mechanism advantages. The input of the model is the original CT image cube centered on the nodule. The degree of malignancy is regarded as the main task, and semantic characteristics are regarded as the branch task. The primary task explains what the SCCNN model learns from the raw image data, and trains it to improve the prediction of whether a nodule in a CT image is malignant.

The contributions of this paper are as follows:

1. We synthesized the semantic characteristics of lung nodules commonly used by doctors during clinical diagnosis to design a multi-task learning network model to assist in identifying benign and malignant nodules, which was experimentally verified to improve the model’s performance and increase the interpretability of the model.
2. A multi-perspective approach is used for data augmentation, combining physician-annotated data and gold-standard pathological diagnostic data to deal with uncertain nodules in order to solve the problems of too little annotated data and sample imbalance and maximize the use of existing annotation information.
3. Establish ablation experiments by introducing different attention mechanisms so that the model can adaptively focus on more critical feature information when synthesizing multiple semantic characteristics and improve the robustness of the model.

The remainder of this paper is organized as follows. Section 2 presents the materials and methods used in this study, including the dataset, data processing methods and the proposed SCCNN model. Section 3 describes the performance evaluation indicators and model results. Section 4 discusses the strengths and limitations of this work. Finally, in Section 5, the conclusions of the study are provided.

## 2. Materials and Methods

### 2.1. Dataset and Data Cleaning

This study used the publicly available Lung Image Database Consortium and Image Database Resource Initiative (LIDC-IDRI) [30] as the underlying data source, which contains 1018 lung CT cases from 1010 lung cancer patients. The CT cases contained lesion annotations from four experienced chest radiologists. The CT images were given digital imaging and communications in medicine (DICOM) format. The annotation results were presented in Extensive Markup Language (XML) format, and the annotations included large nodules (diameter ≥ 3 mm), small nodules (diameter < 3 mm), and non-nodules, with additional identifiers of specific contours and features for large nodules. The four physicians labelled the images with the following nine semantic characteristics: subtlety, lobulation, spiculation, sphericity, margin, texture, internal structure, calcification and malignancy. All of the categories were divided into five grades to differentiate the degree of expression of the semantic characteristics. The calcification was classified in six grades. Evaluating an isolated pulmonary nodule’s specific morphology can help differentiate between benign and malignant nodules. For example, lobulated outlines or spiculation edges are usually associated with malignancy. The presence and pattern of calcification can also help distinguish between the two [31]. These image features provide a quantitative and objective way to capture image information to create more standardized rating systems and terminology, and reduce competent variability between radiologist annotations [32]. Typically, a higher rank indicates a more significant corresponding semantic characteristic. Table 2 shows the specifics of each rank.

LIDC-IDRI provides the diagnostic data of benign-malignant at two levels, semantic benign-malignant and pathological benign-malignant, where the former is mainly based on the information labelled by doctors, i.e., doctors’ subjective judgment of lung nodules based on rich clinical experience, and the latter is based on pathological diagnostic information, i.e., the diagnosis of the nodules through tissue sections and puncture biopsy procedures, which is the “gold standard” for assessing the risk of lung cancer [33].

**Figure 2:**
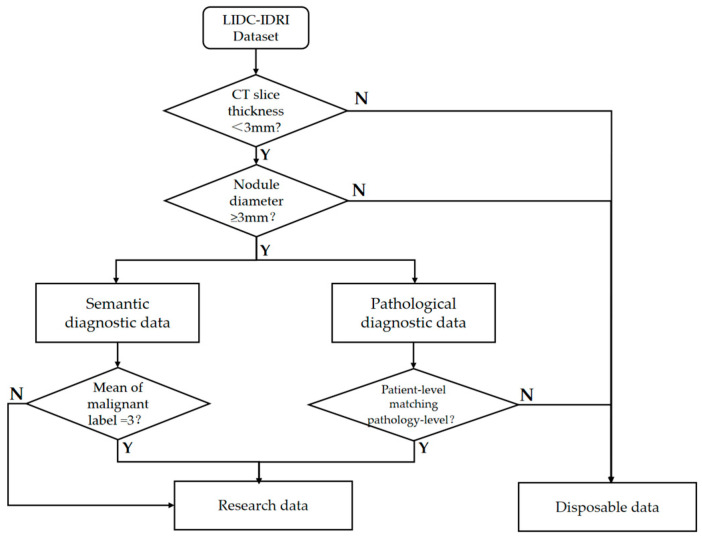
Data cleansing flowchart. Y stands for Yes, a case where the condition is valid, and N stands for No, implying a case where the condition is not valid.

The gold standard data makes it more difficult to obtain data samples because it involves invasive surgical operations and 157 subjects in LIDC had pathologic diagnostic information. There were 2072 lung nodules with physician annotations of semantic characteristic grade in LIDC, each containing annotations from at least one physician. We first screened out high-quality data from these CT imaging data with a nodule diameter greater than 3 mm and CT slice thickness within 3 mm, referring to the positive and negative sample definition method in a previous study [34]. In order to maximize the use of limited annotations for samples of uncertain malignant samples, we added them to the dataset after referring to the pathological diagnosis. Figure 2 shows the specific data cleaning process, and the cleaned data totaled 1779 cases.

### 2.2. Data Preprocessing and Correlation Analysis

In order to quantify the intrinsic association between semantic characteristics and the degree of malignancy, our study performed a Spearman’s correlation [35] analysis on the cleaned data, using the absolute value of the correlation coefficient as a measure of the degree of linear correlation between the two.

The semantic characteristics of the lung nodules with higher correlation can be interpreted as follows: when this characteristic has a higher rank, it may be accompanied by a more pronounced degree of malignancy, which is more likely to enhance the prediction of malignancy by the network model. As can be seen from Table 3, the three characteristics with the highest rank of relevance are lobulation, spiculation and subtlety, indicating that these semantic characteristics play an essential role in malignancy classification [36]. Therefore, they are selected as auxiliary tasks for the model.

Meanwhile, the cleaned data samples are divided into training and test sets in the ratio of 9:1, as shown in Table 4. During the training process, a 10% portion is taken as validation set, so the original dataset is divided into the training set, validation set, and test set corresponding to 81%, 9% and 10% of the divisions. The test set was used as an independent validation and was not involved in data balancing and amplification operations. It was left as it was to show the original proportions of positive and negative samples in the dataset.

The Hounsfield unit (HU) in the CT image reacts to the degree of tissue absorption of X-rays, and the image needs to convert to the HU value before making the window width and window position adjustment [37,38].

Therefore, the CT slices were firstly unified in the range of [−1000, 400]. Then, the CT values of the lung nodule images were extracted and normalized using linear transformation to make the images more transparent. At the same time, since the images in the dataset come from different devices, in order to truly reflect the imaging size of the lung nodules, the voxel spacing in the *x*, *y* and *z* directions is resampled to 1 mm × 1 mm × 1 mm. Controlling the resolution of the input images to be of the same size creates the conditions for the subsequent model to capture the features.

In the study of A.P Reeves [34], the nodal coordinate positions and nodal size and diameter reports were provided, from which the center of mass coordinates were obtained as distance information from the center scanning point of each image. The coordinates obtained by coordinate conversion through Equations (1)–(3) were required to localize the nodule to the image:

(1) $$x^{\prime }=Coord_{x}\cdot OrigSpa_{x},$$

(2) $$y^{\prime }=Coord_{y}\cdot OrigSpa_{y},$$

(3) $$z^{\prime }=(OrigSize_{z}-Coord_{z})\cdot OrigSpa_{z},$$

In the above equation, $Coord_{x}$, $Coord_{y}$ and $Coord_{z}$ represent the original distance information of the image in x, y and z axes, $OrigSpa_{x}$, $OrigSpa_{y}$ and $OrigSpa_{z}$ represent the pixel resolution of the image before resampling, and $OrigSpa_{z}$ represents the size of *z*-axis before resampling. The product $x^{\prime }$, $y^{\prime }$, and $z^{\prime }$ results represent the voxel coordinates of each image in the corresponding direction after a coordinate transformation.

After locating the position, the appropriate size of the Region of Interest (ROI) of the lung nodule will be selected. According to the study [34] and statistics after data cleaning, the diameter of the large nodule in the cross-section of the CT sequence is distributed between 3.06–38.14 mm. In order to encompass all the nodules, the final setting of this study cropped out the 40 × 40 size of the region of interest while setting nine layers. The 3D stereoscopic nodule images with a length and width of 40 mm each and a height of 9 mm were organized.

On the other hand, nine semantic characteristic ratings of nodules were extracted by parsing the XML annotation file. At least one doctor reviewed and annotated each case of CT concerning Shen’s method [13]. When more than one doctor annotates a nodule, the average of the ratings of the multiple doctors was taken and then binarized to serve as a Ground Truth Label (GTL).

The specific way is that when the rating score of 3 is regarded as uncertain samples, the average score below 3 is labelled as benign positive samples with low malignant suspicion, and higher than 3 is labelled as malignant negative samples with high malignant suspicion. In order to maximize the use of the existing labelling, unlike other studies that directly discarded uncertain nodules, this paper refers to the pathological information for further control. We take those that contain the “gold standard” results as training labels. In this way, we labelled an additional 80 samples, of which 30 were benign and 50 were malignant.

Acquiring pathological diagnostic data requires traumatic and costly surgical operations, making it difficult to obtain, and the amount of data is small. We selected samples and screened them to maximize the use of annotation. Furthermore, semantic characteristics data distribution is exceptionally unbalanced, compared with the number of the majority of the categories in their respective attributes, which is too small to meet the needs of data division and subsequent experiments, so binarization is adopted to overcome data sparsity. Table 5 shows the results. Label 0 indicates positive samples that correspond to the semantic characteristics that are not apparent, and label 1 indicates negative samples that the semantic characteristics are evident.

Since the quality and quantity of data limits model training, data augmentation is required to augment the data volume to reduce overfitting. Prior to this, data balancing operations were performed. Specifically, based on Table 3, this study was finally carried out using the on-the-fly pan, rotate and flip technique. The balanced benign samples were 2118 cases, and the malignant samples were 2078 cases. After that, data enhancement was done on this basis. Numerous techniques are explored in existing studies, such as basic, deformable, deep learning or other data augmentation techniques [39]. In our paper, it is due to the tendency to simulate the doctor’s full range of diagnostic state. It is proposed to use the multi-view technique as an augmentation method. The 3D cubes of lung nodules were based on axial, sagittal and coronal planes and rotated by 45 degrees on the coordinate axis to generate nine viewing angles as observation angles. The total amount of augmented lung nodule data reached 37,764 cases [40].

### 2.3. Semantic Characteristic Convolutional Neural Network

In the actual diagnostic process, to observe lung nodules in CT images from multiple perspectives, radiologists can usually select different planes on the computer to understand the morphology and distribution of the nodules. As a three-dimensional imaging technique, the scanning angle of CT can be adjusted according to the needs, and multiple scans can also be performed to understand the growth changes of the nodules. However, for images from different patients with different devices in LIDC-IDRI, the orientation in medical imaging is not fixed for all CT maps. Therefore, extracting nine views of the ROI region for fusion to be used as data input can be used to simulate further diagnosis by doctors using multiple views and maximize the extraction of information around lung nodules with complex shapes.

**Figure 3:**
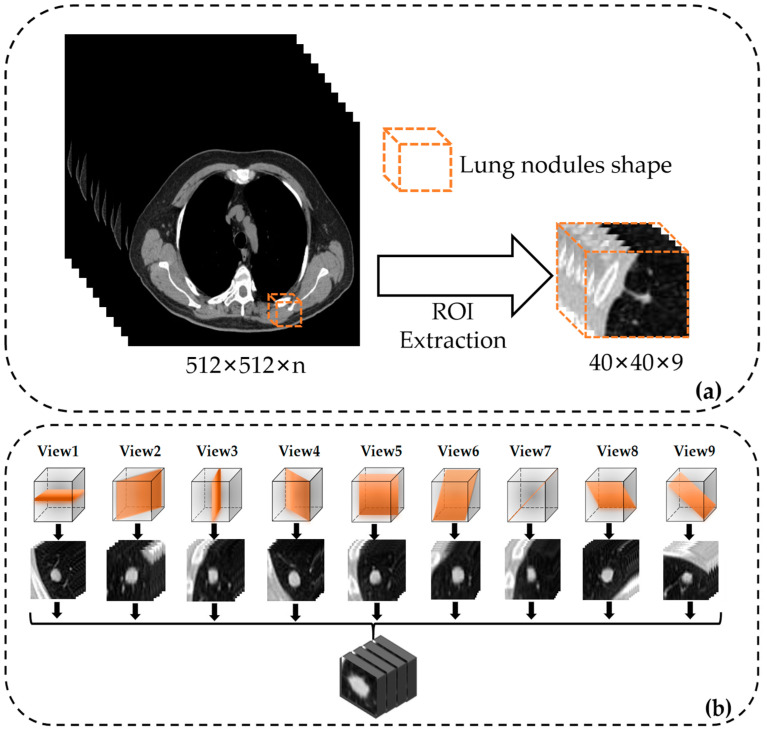

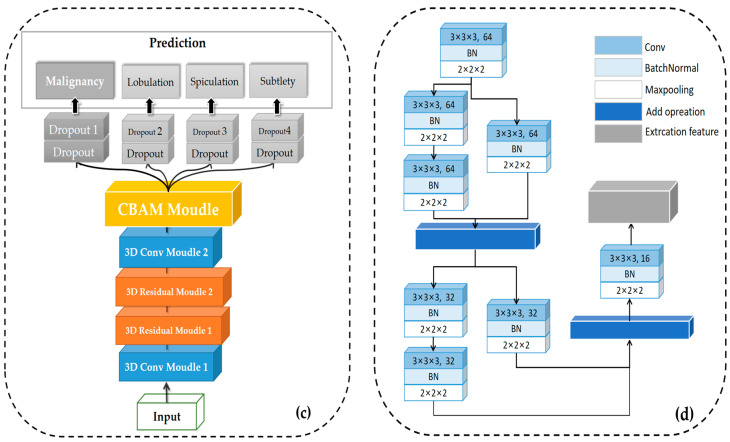
Model architecture of our semantic characteristic convolutional neural network. (**a**) Extraction of ROI from CT images, (**b**) multi-view lung nodule samples, (**c**) Our SCCNN-CBAM model structure and (**d**) Architecture for feature extraction.

As shown in Figure 3, the benchmark network model in this paper consists of a series of convolutional layers, pooling layers and corresponding fully connected layers. The input data is a 40 × 40 × 9 multi-view lung nodule 3D cube, which passes through the first convolutional layer consisting of 64 convolutional kernels of size 3 × 3 × 3. The second and third layers are residual modules [41] consisting of 32 convolutional kernels of size 3 × 3 × 3, and the fourth convolutional layer consists of 16 convolutional kernels of size 1 × 1 × 1. Immediately, the convolutional images are fed again into the kernel of the 2 × 2 × 2 maximum pooling layers, which enters into four fully connected layers after spreading processing to map the extracted features to the output space. The specific calculation is as follows:

(4) $$X_{i}^{l}=\sum_{j}W_{ij}^{l}\cdot X_{j}^{l-1}+b_{i}^{l},$$

where $X_{i}^{l}$ denotes the $i^{th}$ output feature mapping in layer l, $X_{j}^{l-1}$ denotes the $j^{th}$ input feature mapping in layer *l* − 1, $W_{ij}^{l}$ denotes the 3D convolution kernel for connecting sums in layer *l*, ∗ denotes the convolution operation, and $b_{i}^{l}$ denotes the $i^{th}$ bias term in layer *l*. Rectified linear units (ReLUs) [42] are used as activation functions after convolution. It is the fully connected layer that plays the role of classification in the network.

(5) $$X_{f}=W_{f}X_{f-1}+b_{f},$$

In this equation, $W_{f}$ denotes weighting matrix, and $X_{f-1}$ denotes the neuron vector of the previous layer of the fully connected layer, and $b_{f}$ is the bias term for this layer, setting up a dropout layer after the full connectivity layer, which can improve network generalization [43]. The model structure is shown in Figure 3 below.

Semantic characteristics as a focus for radiologists and studies have shown that high-accuracy predictions can be achieved using only semantic characteristics as inputs [41]. In this paper, we first try to add lobulation labels as an auxiliary task and then continue adding spiculation and subtlety. Multiple classification tasks of malignancy degree and semantic characteristics are processed simultaneously to form a multi-task learning model (MTL). The correlation between the tasks is considered in the multi-task learning process. Additional fully connected layers and SoftMax activation functions are added to the underlying network architecture to accommodate the added feature classification tasks, and the Shared-Bottom (SB) is used to share the information about goodness and malignancy and different semantic characteristics extracted during propagation, which ultimately improves the performance of malignancy classification [44]. MTL needs to consider the correlation and weight assignment between tasks. To jointly optimize the SCCNN during the network training, a global loss function is proposed to maximize the probability of predicting the correct label for each task.

(6) $$Loss_{gobal}=\sum_{t=2}^{t\in [2,4]}\lambda_{t}Loss_{t},$$

In this equation, *t* is the $t^{th}$ subtask, which is determined by the number of semantic characteristics that t∈[2,4]. *t* ∈ [2,4] denotes that it takes on integers from 2 to 4. When *t* = 1, it represents that the base CNN model only considers one factor, benign or malignant. When *t* = 2, it represents that the model uses lobulation as a secondary task for classification. When *t* = 3, it represents that the two semantic characteristics, lobulation and spiculation, are used as secondary tasks at the same time. When *t* = 4, it represents that the three semantic characteristics of lobulation sign, spiculation sign and subtlety are considered simultaneously and they are used as a secondary task to assist in benign-malignant classification. $Loss_{t}$ corresponds to a separate loss function for the $t^{th}$ task, which is the individual loss corresponding to the $t^{th}$ task. $\lambda_{t}$ is the weight hyperparameter for the $t^{th}$ task. It is based on the importance of the task in the total loss, and auxiliary tasks exist to better serve the main task. Higher weights are assigned to malignant classification tasks, as they are the result of being given greater expectations. Each loss component is defined as a weighted cross entropy loss in the following equation:

(7) $$L=-\frac{1}{N}\sum_{i}^{N}\sum_{j=1}^{M}y_{i,j}log(p_{i,j}),$$

where *N* is the number of samples in a batch size and M is the number of categories. $y_{i,j}$ is the true label for the $i^{th}$ sample in the $j^{th}$ class; it equals 0 or 1 here. $p_{i,j}$ represents a prediction score for class $j^{th}$. The probability distribution was transformed using the softmax function. The global loss function is minimized during the training process by iteratively computing the gradient of $Loss_{gobal}$ over the learnable parameters of SCCNN and updates the parameters through back-propagation.

### 2.4. Attention Mechanisms

As the scale of tasks increases, there may be interactions between malignancy and semantic characteristics. We hope the model can automatically learn the dependencies between tasks and play the role of a carrier, just like an actual radiologist in the hospital. The Convolutional Block Attention Module (CBAM) [45] is introduced in the stage of feature extraction and fusion to improve the model performance.

The module has two sequential sub-modules, channel and spatial. Specifically, it sums the weight vectors of the sequential outputs of the two sub-modules. After normalization by sigmoid, it obtains a heat map of the same size as the input feature map. Multiply it with the input feature map to get the adjusted feature map.

The goal of channel attention focusing is to find more meaningful features of the input image. Squeezing the spatial dimension of the input feature map can help it to compute the channel attention efficiently. By using both global average-pooling and max-pooling, it learns the relationships between the channels. Subsequent input into the two fully connected layers recalibrate the channel feature responses to learn global information.

(8) $$M_{c}(F)=\sigma (W_{1}\cdot (W_{0}(F_{avg}^{c}))+W_{1}\cdot (W_{0}(F_{max}^{c}))),$$

$F_{avg}^{c}$ and $F_{max}^{c}$ denote the descriptors of the input feature mappings after average-pooling and max-pooling operations, which subsequently forward to the shared network. $W_{0}$ and $W_{1}$ represent the weights of the shared network consisting of a multi-layer perceptron (MLP) with one hidden layer. σ denotes the sigmoid activation function. Eventually an element-wise summation is performed to merge the output feature vectors to produce $M_{c}(F)$, which is the channel attention map.

The target of spatial attention focuses on the information between different locations in the feature map. Since the size of the nodules ranges from 3 to 38 mm, an image with a length and width of 40 mm is used in this study, making the lung nodule present in the surrounding structural organization. The spatial attention module can consider the spatial location, thus focusing on the target region, which operates by performing the maximum pooling and average pooling operations on the feature maps output from the previous step as well and selecting a 7 × 7 × 7 convolution kernel as the attention fuser and dimensionality reduction. $M_{s}(F)$ denotes the spatial attention mapping. The formula can be expressed as:

(9) $$M_{s}(F)=\sigma (f\cdot ([F_{avg}^{c};F_{max}^{s}])),$$

Finally, the output is fused with the original input for feature fusion with the following equation:

(10) $$F^{\prime }=[M_{s}(M_{C}(F)⊗F)]⊗(M_{C}(F)⊗F),$$

**Figure 4:**
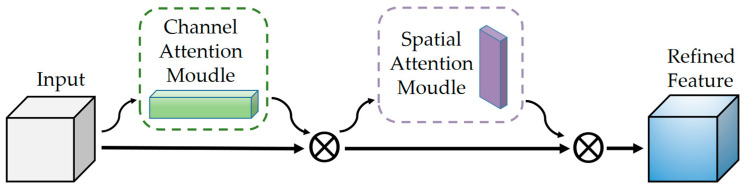
CBAM module structure diagram.

*F* denotes the original input image feature and ⊗ denotes the multiplication operation. The mapping $M_{C}(F)$ acquired in the channel attention multiplies with *F* as input to spatial attention and iterated, and their result $F^{\prime }$ realizes the combination of channel and spatial attention. Figure 4 illustrates the structure of the attention mechanism. 

## 3. Results

### 3.1. Evaluation Metrics

This study used the GPU NVIDIA Quadro K2200M to establish the experimental environment. The deep learning model was implemented in Python 3.6 using the TensorFlow 1.9.0 and the Keras 2.1.6 toolkit. The Adam optimizer was used for the backpropagation updating during the model training process [46]. This study used the early stopping strategy to optimize the learning rate and prevent model overfitting, and the patience is 10. We set the initial learning rate to 0.01, and the minimum learning rate was 1 × 10^−6^. The mini-batch size was 64 after our many experiments.

To evaluate and compare the SCCNN effect on lung nodule malignancy prediction, an ordinary 3D CNN was first implemented as a baseline model. Then, we used four metrics of accuracy (Acc), specificity (Spe), sensitivity (Sen), and the area under the receiver operating characteristic (ROC) curve. They are defined as follows:

(11) $$Acc=\frac{TP+TN}{TP+TN+FP+FN},$$

(12) $$Sen=\frac{TP}{TP+FN},$$

(13) $$Spe=\frac{TN}{TN+FP},$$

(14) $$Precision=\frac{TP}{TP+FP},$$

(15) $$Recall=\frac{TP}{TP+FN},$$

(16) $$F1=2\times \frac{Precision\times Recall}{Precision+Recall},$$

where *TP*, *TN, FN*, and *FP* denote true positive (true positive, TP), true negative (true negative, TN), false positive (false positive, FP), and false negative (false negative, FN), respectively, TP and TN represent the number of positive and negative samples that a correctly classified, and FN and FP refer to the number of positive and negative samples that were misclassified. ROC, as the curve formed by the true positive and false positive rates, is a composite of *Sen* and *Spe*. The value of AUC assesses the credibility of the classifier, and the value is proportional to the credibility.

### 3.2. Prediction Results of Models

The following Table 6 shows the metrics comparing SCCNN versus basic 3D CNN performance.

Through the visual inspection of the table, it can be seen that the accuracy of CNN is 89.77%, which has the single task of benign-malignant classification. SCCNN reached 93.75% when it synthesized lobulation. It reached 93.18% after synthesizing lobulation and the spiculation of semantic characteristics. It has achieved 94.89% when synthesizing lobulation, spiculation, and subtlety. Compared with CNN, it has improved by five percentage points, and AUC has also improved from 0.924 to 0.966. At the same time, the model can additionally output the classification results of the semantic characteristics. The metric assessments show that the proposed SCCNN achieved better performance for malignancy prediction compared with the CNN approach. Table 7 shows the prediction results for semantic characteristics. Due to their more unbalanced data distribution, Precision and F1 scores were used as evaluation metrics. These results denote that the SCCNN model is able to learn feature representations that are predictive of semantic characteristics while simultaneously achieving high performance in predicting nodule malignancy.

In addition, SCCNN was introduced with the CBAM attention module to automatically focus on the more critical characteristics due to the increased amount of information. It was conducted on SCCNN separately. Table 8 shows the results.

As can be seen, the AUC of the model synthesizing the three semantic characteristics improved, rising to 0.973, and the ACC improved, rising to 95.45%, which is the best performance. However, the metrics of SCCNN I decreased slightly, indicating that the attention mechanism is more suitable for information-rich situations. When the input data contains richer features, the attention module is able to fuse and focus on more valuable information, effectively improving performance. However, in some cases the additional calculations and parameters brought by themselves may increase the risk of model overfitting and add unnecessary complexity, so it is necessary to consider the task requirements and model complexity comprehensively.

**Figure 5:**
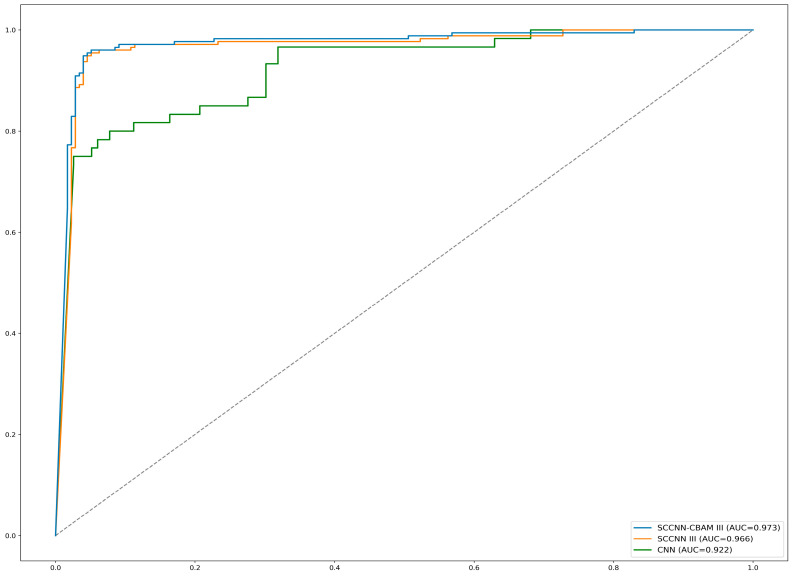
Receiver operating characteristic curve comparison: SCCNN-CBAM versus SCCNN versus 3D CNN. (SCCNN III was chosen to represent a model represented by a fusion of three semantic characteristics: lobulation, burr, and subtlety).

Figure 5 shows the receiver operating characteristic (ROC) curve plots comparing SCCNN-CBAM versus SCCNN versus 3D CNN performance. These plots represent the intuitive trade-off between sensitivity and specificity. Through the visual inspection of the ROC curves, it can be seen that SCCNN performs better than the basic CNN model.

## 4. Discussion

### Existing Studies

We present the model named SCCNN which can incorporate specialized field knowledge of medical imaging into the model architecture, predicting semantic characteristics along with the primary task of nodules malignancy diagnosis. The following three strongly related semantic characteristics were mainly considered: lobulation, spiculation and subtlety. Our results in Section 3 show that it finishes the job of classification efficiently.

As shown in Table 9, the method proposed in this study was compared with the existing methods.

Shen et al. [47] utilized a hierarchical design to incorporate semantic features into a convolutional neural network to complete the classification of five semantic features in the low-level task component. The high-level benign and malignant classification task was accomplished by fusing the features extracted from the penultimate generative layer. However, the five features covered the data imbalance, which may have affected the results. Zhai et al. [48] extracted nine directional nodal views and used multitask learning to perform benign and malignant classification and image construction tasks. However, semantic features were not considered, and the images stayed at the 2D level. The study by Liu et al. [49] had lower Acc and Sen values than this study. However, the AUC was slightly higher than this study because it adopted all of the semantic features provided in the LIDC and set up the regression module for the features, which resulted in higher complexity of the model.

**Figure 6:**
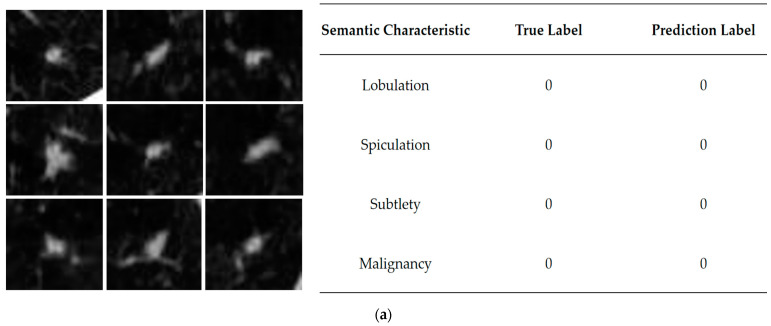

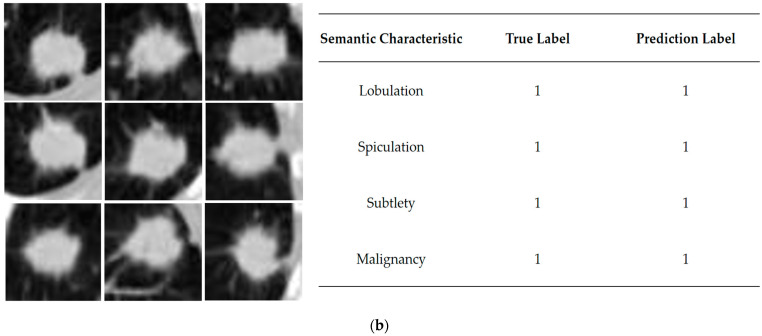
Interpretability analysis of SCCNN prediction results, and it demonstrates a situation where the prediction is correct. (**a**) The left side shows slices of the 9 views corresponding to the benign nodule. The right side shows their corresponding semantic characteristics and malignancy levels are on the right side for predictive and actual labels; (**b**) the left side shows slices of the 9 views corresponding to the malignant nodule. The right side shows their corresponding semantic characteristics and malignancy levels are on the right side for predictive and actual labels.

The study takes the double case of benign and malignant nodules to demonstrate the semantic characteristics and the visualization of benign-malignant results. It reflects the interpretability of SCCNN in this way. Figure 6 illustrates the case of correct prediction. As shown in Figure 6 below, the left side in Figure 6a shows the center slice visualization image of a benign nodule in each of the nine views. The SCCNN correctly predicts it as benign. The nodule sample seems to have rounded edges, with no stark contrast to the surroundings, and no obvious lobulation or spiculation signs. The predictive and true labels were the same, consistent with the judgment of a physician with specialized domain knowledge.

Figure 6b shows the nodule predicted to be malignant by the model. It is also predicted to have obvious lobulation, spiculation signs and clear contrast with the environment. The image also shows that the edges are sharp and easy to observe. This prediction also explains to some extent why the model combined with the domain knowledge of semantic characteristics predicts it as malignant. The aim is to simulate the process of diagnosing a nodule by a radiologist. Compared with the model that directly outputs benign and malignant results, it provides more substantial explanatory and convincing results and overcomes the black-box problem to a certain extent.

**Figure 7:**
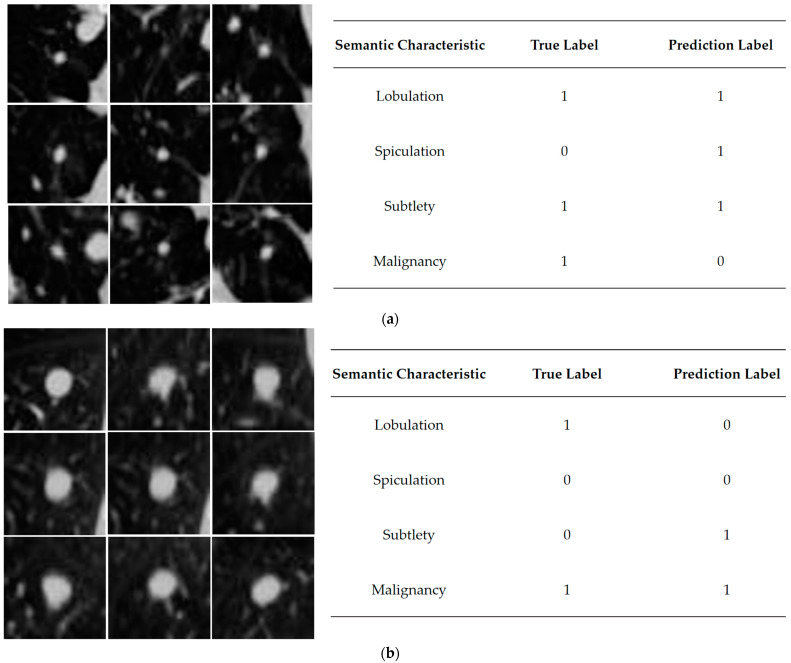
Semantic characteristics or benign and malignant errors in SCCNN prediction. (**a**) An example of a benign nodule with a successful malignant label prediction but two semantic characteristics incorrectly predicted; (**b**) An example of a malignant nodule with a successful prediction of the malignant label but an incorrect prediction of two semantic characteristics.

Next, Figure 7 shows examples of the model’s prediction errors on tasks either the semantic characteristic classification task or the benign and malignant classification task. Figure 7a shows that the model predicts malignant samples as benign nodules but correctly predicts a portion of semantic characteristics. Figure 7b shows that the model predicts proper labels in malignancy but incorrectly in lobulation and subtlety. The reasons for this may be that although the nodule appears smooth and round-like in one view, the multiple views cause the shape displayed in several other views to be more elongated; the nodule’s surroundings are complex, and it implicated in fine blood vessels or surrounding tissue structures; and the semantic characteristics selected for the study are limited help in terms of number and grading for malignancy determination. The semantic labels are beneficial in interpreting the model’s predictions for malignancy, mapping the features used by the network for the benign-malignant prediction task to establish domain knowledge about lung nodules.

We also summarize the shortcomings of this study. Firstly, it is due to the imbalance between classes. The data distribution of some semantic characteristics is seriously imbalanced (e.g., calcification, internal structure), leading us to select only those with high relevance as the object of study. Second, some semantic characteristics have very few dependent subclass samples, also known as an intra-class imbalance. We had to binarize the labels from their own five or six classes. Such an operation may cause some information to be lost, and the essence is that the amount of medical image labelling is scarce and difficult to obtain. It is also a common obstacle in developing medical image processing in artificial intelligence. Finally, radiologists may also need to combine the patient’s age, medical history, regular review results and other images when diagnosing lung nodules. In future, this study will consider further data collection for model optimization, such as the dynamic changes of the same nodule at different periods, multimodal examination results, etc. It will assist the development of deep learning technology in lung nodule classification.

## 5. Conclusions

In this study, we propose a new SCCNN model that synthesizes semantic characteristics for benign and malignant classification of lung nodules. The model uses semantic characteristic prediction as a branched (auxiliary) task to improve the accuracy of predicting the malignancy of nodule samples in CT images. It also outputs predictions of three semantic characteristics. SCCNN allows the shared convolutional module to learn generalizable features between related tasks, extensively validated on the open-source dataset LIDC-IDRI. The introduction of multiple views of CT images and an attention mechanism also brings the scenario closer to reality. The interpretability of the model helps clinicians to make further judgments. In future work, we hope to obtain a more valuable dataset, including nodal images and richer semantic characteristics, and to delineate a more detailed classification hierarchy.

## Figures and Tables

**Table 1:** Summary of the contribution and development of existing research in the field.

Existing Research	Highlights	Contribution
Shen et al. [13]	Multiple scales	Simplify the traditional way
Liu et al. [14]	Integrated learning	Simulate different expert behaviors
Ciompi et al. [15]	Multi-view(three views)	A multi-scale representation using three scales
Zheng et al. [16]	Scale-shifting module	Scaling the images with different resolutions
Wu et al. [22]	Combine lung nodule segmentation, semantic and malignant prediction	Improve a single task’s performance
Zhao et al. [23]	Multi-scale and multi-task	Combined two image features of different volume
Li et al. [24]	Domain knowledge into CNN	Achieved the classification of nine semantic characteristics
Zhang et al. [27]	Both spatial and channel dimensions	Improving detection effectiveness
Fu et al. [28]	Attention module	Compute the importance of each slice
AI-Shabi et al. [29]	3D axial attention	Applying the attention to each axis individually

**Table 2:** Grading of semantic characteristic.

Semantic Characteristics	1	2	3	4	5	6
Subtlety	Extremely subtle	Moderately subtle	Definitely subtle	Moderately distinct	Distinct	-
Internal structure	Soft tissue	Fluid	Fat	Air	-	-
Calcification	Popcorn	Laminated	Solid	Non-central	Central	None
Sphericity	Linear	-	Ovoid	-	Round	-
Margin	Vague	-	-	-	Sharp	-
Lobulation	None	-	-	-	Distinct	-
Spiculation	None	-	-	-	Distinct	-
Texture	Non-solid	-	Mixed	-	Solid	-
Malignancy	Highly unlikely	Moderately unlikely	Indeterminate	Moderately suspicious	Highly suspicious	-

- There is not precisely expressed in the LIDC for the corresponding level.

**Table 3:** Correlation analysis of each semantic characteristic with malignancy.

Semantic Characteristics	Correlation Coefficient
Lobulation	0.494 **
Spiculation	0.376 **
Subtlety	0.348 **
Margin	−0.305 **
Calcification	0.280 **
Texture	−0.177 **
Sphericity	−0.173 **
Internal structure	0.083 **

** *p* < 0.01.

**Table 4:** Training set and test set sample division.

Datasets	Benign Nodules Samples	Malignant Nodules Samples
Train dataset	1058	544
Test dataset	117	60

**Table 5:** Data segmentation of semantic characteristics after binarization.

Datasets	Label 0	Label 1
Lobulation	1424	355
Spiculation	1516	263
Subtlety	547	1232

**Table 6:** Performance comparison between original 3D CNN and SCCNN after stepwise synthesizing highly relevant semantic characteristics. The bold value represents the optimal result based on the same metrics.

Model Types	AUC	Acc	Spe	Sen
CNN ^1^	0.924	89.77%	89.65%	90.0%
SCCNN I ^2^	**0.971**	93.75%	93.82%	91.66%
SCCNN II ^3^	0.959	93.18%	92.24%	93.0%
SCCNN III ^4^	0.966	**94.88%**	**94.82%**	**95.0%**

^1^ CNN here refers to the base model that performs only benign and malignant classification task. ^2^ I-SCCNN refers to SCCNN introduces lobulation on the basis of malignancy, totaling one semantic characteristics. ^3^ II-SCCNN refers to SCCNN introduces lobulation and spiculation, totaling two semantic characteristics. ^4^ III-SCCNN refers to SCCNN introduces lobulation, spiculation and subtlety, totaling three semantic characteristics. Bolded data in the table indicate the highest value achieved under the indicator.

**Table 7:** Classification performance for semantic feature predictions.

Semantic Characteristics	AUC	Acc	Pre	F1-Score
Lobulation	0.884	92.04%	88.66%	87.11%
Spiculation	0.853	93.75%	93.13%	82.78%
Subtlety	0.691	89.77%	83.75%	77.44%

**Table 8:** Results of models introduced CBAM attention module. The bold value represents the optimal result based on the same metrics.

Model Type	AUC	Acc	Spe	Sen
SCCNN-CBAM I	0.956	93.18%	92.24%	95.0%
SCCNN-CBAM II	0.957	92.05%	91.38%	93.33%
SCCNN-CBAM III	**0.973**	**95.45%**	**94.83%**	**96.66%**

The bolded data in the table indicate the highest value achieved under the indicator.

**Table 9:** Performance comparison between this research method and existing research methods. The bold value represents the optimal result based on the same evaluation metric of the same task.

Method	AUC	Acc	Spe	Sen	Data Source	Semantic Characteristics
Shen et al. [47]	0.856	84.2%	88.9%	70.5%	LIDC	Five (calcification, margin, subtlety, texture, and sphericity)
Zhai et al. [48]	0.9559	N/A	88.87%	87.74%	LIDC	Not involved
Liu et al. [49]	0.979	93.5%	89.4%	93.5%	Private data	All nine semantic characteristics
Our method	0.973	95.45%	94.83%	96.66%	LIDC	Three (lobulation, spiculation, subtlety)

## Data Availability

The dataset are available on reasonable requests from corresponding author.
